# Metabolite profiling in two contrasting Tibetan hulless barley cultivars revealed the core salt-responsive metabolome and key salt-tolerance biomarkers

**DOI:** 10.1093/aobpla/plz021

**Published:** 2019-04-06

**Authors:** Yulin Wang, Xingquan Zeng, Qijun Xu, Xiao Mei, Hongjun Yuan, Dunzhu Jiabu, Zha Sang, Tashi Nyima

**Affiliations:** 1 State Key Laboratory of Hulless Barley and Yak Germplasm Resources and Genetic Improvement, Lhasa, China; 2 Institute of Agricultural Research, Tibet Academy of Agricultural and Animal Husbandry Sciences, Lhasa, China; 3 Wuhan Metware Biotechnology Co., Ltd, Wuhan, China; 4 Tibet Academy of Agricultural and Animal Husbandry Sciences, Lhasa, Tibet, China

**Keywords:** Biomarkers, hulless barley, salinity stress, tolerance mechanisms, widely targeted metabolites

## Abstract

Salinity stress represents one of the most harmful abiotic stresses for agricultural productivity. Tibetan hulless barley is an important economic crop widely grown in highly stressful conditions in the Qinghai-Tibet Plateau and is often challenged by salinity stress. To investigate the temporal metabolic responses to salinity stress in hulless barley, we performed a widely targeted metabolomic analysis of 72 leaf samples from two contrasting cultivars. We identified 642 compounds 57 % of which were affected by salt stress in the two cultivars, principally amino acids and derivatives, organic acids, nucleotides, and derivatives and flavonoids. A total of 13 stress-related metabolites including piperidine, L-tryptophan, L-glutamic acid, L-saccharopine, L-phenylalanine, 6-methylcoumarin, cinnamic acid, inosine 5′-monophosphate, aminomalonic acid, 6-aminocaproic acid, putrescine, tyramine and abscisic acid (ABA) represent the core metabolome responsive to salinity stress in hulless barley regardless of the tolerance level. In particular, we found that the ABA signalling pathway is essential to salt stress response in hulless barley. The high tolerance of the cultivar 0119 is due to a metabolic reprogramming at key stress times. During the early salt stress stages (0–24 h), 0119 tended to save energy through reduced glycolysis, nucleotide metabolism and amino acid synthesis, while increased antioxidant compounds such as flavonoids. Under prolonged stress (48–72 h), 0119 significantly enhanced energy production and amino acid synthesis. In addition, some important compatible solutes were strongly accumulated. By comparing the two cultivars, nine salt-tolerance biomarkers, mostly unreported salt-tolerance compounds in plants, were uncovered. Our study indicated that the salt tolerant hulless barley cultivar invokes a tolerance strategy which is conserved in other plant species. Overall, we provide for the first time some extensive metabolic data and some important salt-tolerance biomarkers which may assist in efforts to improve hulless barley tolerance to salinity stress.

## Teaser

Tibetan hulless barley is an important economic crop widely grown in highly stressful conditions in the Qinghai-Tibet Plateau and is often challenged by salinity stress. We investigated the temporal metabolic responses to salinity stress by employing the widely targeted metabolomic analysis in leaf samples from two contrasting cultivars. We successfully identified 13 stress-related metabolites representing the core metabolome responsive to salinity stress. Moreover, we revealed that the efficient management of glycolysis and energy consumption, especially during the most stressful time is the key strategy for tolerating salt stress. Finally, some unreported salt-tolerance biomarkers were unveiled in hulless barley.

## Introduction

Most of the food and feed crops are salt-sensitive; therefore, salinity stress represents one of the most harmful abiotic stresses for agricultural productivity (Roy *et al.* 2014). Worldwide, over 45 million hectares of irrigated lands have been damaged by salt, and 1.5 million hectares are out of production each year because of the high salinity levels in the soil (Munns and Tester 2008). The vast areas affected and the seriousness of salt affecting agricultural land are predicted to worsen due to an inadequate drainage of the irrigated lands, poor management of the fertilizers applied and global warming (Munns and Gilliham 2015). The primary cause of the salinity stress is an increase in sodium (Na^+^) and chloride (Cl^−^) ions, the most abundant salt ions in natural sources (Shabala and Mackay 2011). High salinity induces several disorders in plants: water stress, ion toxicity, nutritional disorders, oxidative stress, alteration of metabolic processes, membrane disorganization, reduction of cell division and expansion, genotoxicity, etc. (Munns 2002; Flowers 2004; Demidchik *et al.* 2010; Flowers and Colmer 2015). Together, these effects reduce plant growth, productivity and survival. Many plants have evolved several mechanisms for either to exclude salt from the cells or to tolerate its presence within the cells, which include the regulation of ion homeostasis, osmotic adjustment, the regulation of phytohormones, the induction of antioxidant enzyme activity, etc. (Ashraf *et al.* 2008). Gradually, an increasing number of salt-responsive genes have been identified in plants, including ion transporters, free radical scavengers, aquaporins, heat shock proteins, transcription regulators, osmoprotectants and late embryogenesis abundant proteins (Kawasaki *et al.* 2001; Wang *et al.* 2016; Yong *et al.* 2015).

Metabolomics has become a robust approach for plant biologists to understand the complex metabolic responses to various abiotic pressures such as salt stress (Nakabayashi and Saito 2015). Systems biology has mainly focused on studying genomics, transcriptomics and proteomics which only represent the potential for a biological outcome. Unlike the above-mentioned ‘omics’ measures, metabolites and their concentrations directly reflect the underlying biochemical activity and state of cells, tissues or organisms (Kosmides *et al.* 2013). Metabolomics approach has been successfully employed to uncover important metabolites, serving as osmolytes and osmoprotectants, involved in salt stress responses in tomato, *Arabidopsis thaliana*, barley, peanut, alfalfa and safflower (Cramer *et al.* 2007; Zuther *et al.* 2007; Shulaev *et al.* 2008; Widodo *et al.* 2009; Gengmao *et al.* 2015; Song *et al.* 2017; Cui *et al.* 2018).

Tibetan hulless barley (*Hordeum vulgare* var. *nudum*) has developed a strong tolerance system to cope with environmental stresses as compared to other plant species within the genus of *Hordeum* (Li *et al.* 2016). In fact, the crop is widely grown in highly stressful conditions in the Qinghai-Tibet Plateau generally called ‘roof of the world’ because of its extremely high altitude and harsh environment (Dai *et al.* 2012). Therefore, the crop has to suffer various types of abiotic stresses such as cold, drought and salinity (Deng *et al.* 2013), which seriously impair its productivity. As the most important staple food and economic crop for the population in Tibet and its vicinity, it is important to enhance hulless barley stress tolerance for a higher yield and productivity. Unfortunately, to date, only limited progress has been made to enhance the stress tolerance in this crop. More importantly, several studies have focused on drought and cold tolerance enhancement in hulless barley (Deng *et al.* 2013; Zeng *et al.* 2016; Liang *et al.* 2017; Xu *et al.* 2017; Yuan *et al.* 2017) but research on the salinity tolerance has been largely overlooked. The salinization of cropland soil in the Tibetan Plateau has become an increasingly serious concern for agricultural production (Wang *et al.* 2018). It is estimated that 4 % of the total crop land soil is salinized land with an increasing annual rate of 0.15 % (Wang *et al.* 2008). Recently, we launched a breeding programme for hulless barley improvement towards salt tolerance. Over 1700 diverse Tibetan hulless barley accessions were screened for salt-tolerance and some contrasting accessions, including cultivars 0119 and 0226 were identified. The tolerant cultivar 0119 can survive for a week under high salinity stress while the sensitive cultivar 0226 dies just after 4 days. The goal of this study is to get an insight into the dynamic metabolic response to salinity stress and to uncover the major metabolites conferring salinity tolerance, which could be regarded as key biomarkers in hulless barley breeding programmes. To achieve this objective, we extracted, quantified and analysed a large number of metabolites accumulated under progressive salinity stress and we highlighted the divergent metabolic responses of the two contrasting cultivars 0119 and 0226.

## Materials and Methods

### Plant materials and growth conditions

In the present study, two Tibetan hulless barley cultivars 0119 (tolerant to salinity stress) and 0226 (sensitive to salinity stress) were used. They were identified after an initial screening of 1700 accessions. The seeds were surface-sterilized with 10 % (v/v) H_2_O_2_ for 15 min, rinsed thoroughly with distilled water, and then sown in a plastic tray (37 × 35 × 25 cm). The tray was filled with nutritional soil (pH 5.8–6.5, particle size: 0.5–3.0 mm, N: 200 mg L^−1^, PO_3_: 2500 mg L^−1^, K: 200 mg L^−1^ and Mg: 200 mg L^−1^; Nippi Engei Baido No. 1, Nihon Hiryo, Tokyo, Japan): vermiculite (1:1) and kept in a plant incubator set at 25 °C, 2000 µmol m^−2^ s^−1^. At the two-leaf stage, seedlings were removed from the tray and thoroughly washed with tap water. Well-developed and uniform seedlings were selected and transplanted to a 5-L black plastic bucket filled with 4.5 L ½ strength Hoagland nutrient solution (Hoagland and Arnon 1950). Each container was covered with a polystyrol plate with eight evenly spaced holes (one plant per hole) and kept in a growth chamber set at 16–18 °C (with a photoperiod of 18 h light/6 h dark and a relative humidity of 80 %). After 7 days’ growth, the solution was renewed and the corresponding containers were supplemented with 250 mM NaCl. Control plants were maintained in a ½ strength Hoagland nutrient solution without adding NaCl. The experiment was laid in a split-plot design with treatment as the main plot and cultivar as the subplot, and there were five replicates for each treatment. The solution pH was adjusted to 5.8 with NaOH or HCl as required and continuously aerated with pumps throughout the experiment. Then, from the beginning of the stress application, the youngest fully expanded leaves were harvested at 0, 2, 8, 24, 48 and 72 h from the control treatment (CK) and stress treatment (S). Three biological replicates from three different plants were collected at each time point and from each treatment, with a total of 72 samples. Samples were immediately snap-frozen in liquid nitrogen, ground in liquid nitrogen, stored in 15 mL Falcon tubes at −80 °C and later used for metabolomics investigation.

### Widely targeted metabolomics

The sample preparation, extract analysis, metabolite identification and quantification were performed at Wuhan MetWare Biotechnology Co., Ltd (www.metware.cn) following their standard procedures.

### Sample preparation

The freeze-dried leaf samples were crushed using a mixer mill (MM 400, Retsch) with a zirconia bead for 1.5 min at 30 Hz. One hundred (100) mg powder was weighed and aliquots were extracted overnight at 4 °C with 1 mL 70 % aqueous methanol. Following centrifugation at 10 000 g for 10 min, the extracts were absorbed (CNWBOND Carbon-GCB SPE Cartridge, 250 mg, 3 mL; ANPEL, Shanghai, China, www.anpel.com.cn/cnw) and filtrated (SCAA-104, 0.22 µm pore size; ANPEL, Shanghai, China, www.anpel.com.cn/cnw) before the LC-MS analysis (Chen *et al.* 2013).

### HPLC conditions

The sample extracts were analysed using an LC-ESI-MS/MS system (HPLC, Shim-pack UFLC SHIMADZU CBM30A system, www.shimadzu.com.cn; MS, Applied Biosystems 4500 Q TRAP, www.appliedbiosystems.com.cn). The analytical conditions were as follows, HPLC: column, Waters ACQUITY UPLC HSS T3 C18 (1.8 µm, 2.1 mm × 100 mm); solvent system, water (0.04 % acetic acid):acetonitrile (0.04 % acetic acid); gradient programme, 95:5 V/V at 0 min, 5:95 V/V at 11 min, 5:95 V/V at 12 min, 95:5 V/V at 12.1 min, 95:5 V/V at 15 min; flow rate, 0.40 mL min^−1^; temperature, 40 °C; injection volume: 5 μL. The effluent was alternatively connected to an ESI-triple quadrupole-linear ion trap (Q TRAP)-MS.

### ESI-Q TRAP-MS/MS

Linear ion trap (LIT) and triple quadrupole (QQQ) scans were acquired on a Q TRAP-MS, API 4500 Q TRAP LC/MS/MS System, equipped with an ESI Turbo Ion-Spray interface, operating in a positive ion mode and controlled by the Analyst 1.6 software (AB Sciex). The ESI source operation parameters were as follows: ion source, turbo spray; source temperature 550 °C; ion spray voltage 5500 V; ion source gas I, gas II, curtain gas were set at 55, 60 and 25 psi, respectively; the collision gas was high. Instrument tuning and mass calibration were performed with 10 and 100 μmol L^−1^ polypropylene glycol solutions in QQQ and LIT modes, respectively. Based on the self-built database MetWare Database (http://www.metware.cn/) and metabolite information in public database, the materials were qualitatively analysed according to the secondary spectrum information and the isotope signal was removed during the analysis. QQQ scans were acquired as multiple reaction monitoring (MRM) experiments with collision gas (nitrogen) set to 5 psi (Fraga *et al.* 2010). De-clustering potential (DP) and collision energy (CE) for individual MRM transitions were done with further DP and CE optimization (Chen *et al.* 2013). A specific set of MRM transitions were monitored for each period according to the metabolites eluted within this period.

### Metabolomics data analysis

Data matrices with the intensity of metabolite features at the six time points under both salt and control conditions for the two cultivars were uploaded to the Analyst 1.6.1 software (AB SCIEX, Ontario, Canada). For statistical analysis, missing values were assumed to be below the limits of detection, and these values were imputed with a minimum compound value (Chen *et al.* 2013). The relative abundance of each metabolite was log transformed before analysis to meet normality. A Dunnett’s test was used to compare the abundance of each metabolite between different time points. False discovery rate was used for controlling multiple testing. The supervised multivariate method, partial least squares-discriminant analysis (PLS-DA), was used to maximize the metabolome difference between the control and salt-treated samples, as well as the difference between the two cultivars. The relative importance of each metabolite to the PLS-DA model was checked using a parameter called the variable importance in projection (VIP). Metabolites with VIP > 1.0 were considered as differential metabolites for group discrimination. Principal component analysis (PCA), hierarchical cluster analysis (HCA) and Kyoto Encyclopedia of Genes and Genomes (KEGG) pathway analysis were performed in the R software (www.r-project.org). In addition, the central pathway analysis was performed using the Vanted software v.2.2.1 (Junker *et al.* 2006) for the identified important metabolites using *A. thaliana* pathway libraries.

## Results

### Temporal metabolic profiling in leaves of 0119 and 0226 under salt stress

**Figure 1.**
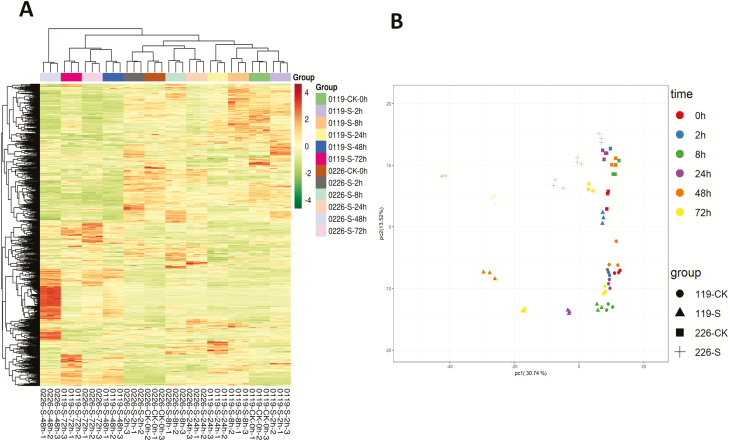
(A) Heatmap hierarchical clustering of detected metabolites. Hierarchical trees were drawn based on detected metabolites in leaves of 0119 and 0226 at 0, 2, 8, 24, 48 and 72 h in control (CK) and salt stress treatment (S). The columns correspond to cultivar at different time points, while the rows represent different metabolites; (B) PCA loading plot of the two first principal components based on the metabolic data from leaves of 0119 and 0226 at 0, 2, 8, 24, 48 and 72 h in control (CK) and salt stress treatment (S).

In the present study, we profiled the metabolic changes in two hulless barley cultivars displaying contrasting responses to salinity stress. In total, we detected 642 metabolites grouped into 32 major classes using the widely targeted metabolomics approach **[see****Supporting Information—Table S1****]**. The majority of the identified metabolites belong to the classes of organic acids, amino acid and derivatives, and nucleotide and its derivative. Conversely, very few metabolites from the classes of catechin derivatives, pyridine derivatives and isoflavone were identified in hulless barley leaves (Table 1). Clustering analysis of the metabolic profiles from the two cultivars showed that all the biological replicates were grouped together, indicating a good correlation between replicates and the high reliability of our data (Cui *et al.* 2018). Moreover, we observed that metabolic data from the early stages of salinity stress (0–24 h) was clearly separated from those from the late stages (48–72 h) (Fig. 1A). Consistently, based on the PCA, a clear separation between the time points could be observed by the PC1. Furthermore, the PC2 distinctly distinguished the two cultivar (Fig. 1B). These results suggest that distinct metabolic programmes are engaged at the early and late stages of salt stress and the differential metabolic responses between 0119 and 0226 could be the basis of their contrasting tolerance to salinity stress.

**Table 1.** Classification of the 642 detected metabolites in hulless barley accessions into major classes.

Class	Number of compounds	Class	Number of compounds
Organic acids	59	Phytohormones	15
Amino acid derivatives	58	Vitamins	14
Nucleotide and its derivates	56	Carbohydrates	13
Flavone	54	Benzoic acid derivatives	11
Flavone C-glycosides	45	Tryptamine derivatives	10
Hydroxycinnamoyl derivatives	32	Flavonolignan	10
Lipids_glycerophospholipids	31	Indole derivatives	9
Amino acids	27	Cholines	7
Others	26	Alkaloids	7
Phenolamides	26	Anthocyanins	5
Quinate and its derivatives	20	Nicotinic acid derivatives	4
Lipids_fatty acids	19	Alcohols and polyols	4
Lipids_glycerolipids	18	Terpenoids	3
Flavanone	18	Catechin derivatives	3
Coumarins	17	Pyridine derivatives	2
Flavonol	17	Isoflavone	2

### Alteration of the metabolite levels induced by salt stress

**Figure 2.**
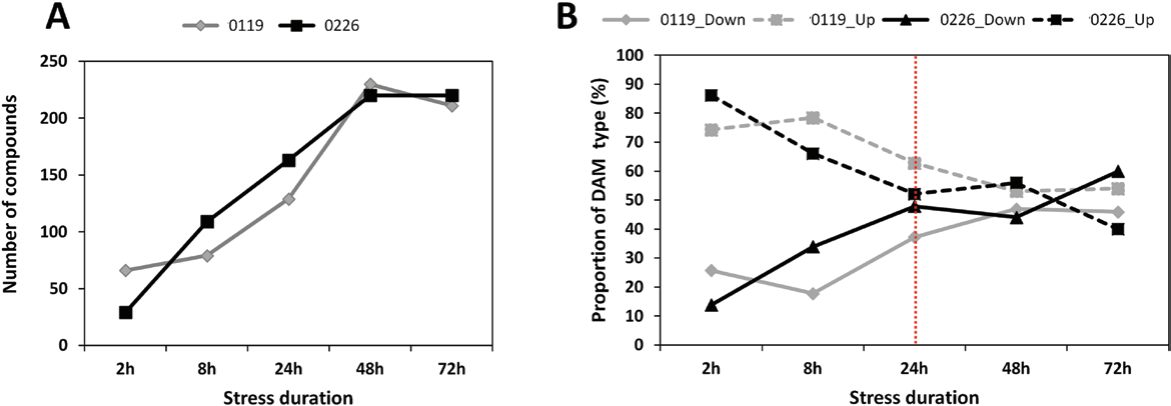
Temporal changes of metabolic reprogramming in 0119 and 0226 under salt stress. (A) DAMs at 2, 8, 24, 48 and 72 h under salt stress; (B) proportion of DAM types (down- and up-accumulated) at 2, 8, 24, 48 and 72 h under salt stress. The red dashed line depicts the critical time point for salt stress endurance in hulless barley.

In order to identify the differentially accumulated metabolites (DAMs) under salinity stress, we compared the metabolite levels in the control condition to their levels under stress at each time point. We identified from 66 to 230 DAMs (364 DAMs in total) in 0119 and from 29 to 220 DAMs (364 DAMs in total) in 0229 at the different time points under stress (VIP > 1) **[see****Supporting Information—Table S2****]**. These salt-responsive compounds are mainly primary metabolites including organic acids, nucleotide and its derivates, and amino acid derivatives. We observed an increasing DAM number over the stress duration in both cultivars, indicating an active metabolic readjustment to respond to the stress (Fig. 2A). However, after 2 h of salt stress, the number of DAMs affected in the sensitive cultivar 0226 was significantly higher than 0119, which denote the strong metabolic perturbation experienced by 0226. By scrutinizing the types of DAM (up- and down-accumulated) in both cultivars during the time points, it could be noticed that the tolerant cultivar responded quickly to the stress by strongly decreasing key metabolites from the class of nucleotides and its derivatives (adenosine 3′-monophosphate, adenosine, inosine 5′-monophosphate, deoxyadenosine) to control the energy homeostasis, while strikingly increased flavonoids (>2300-fold increase of chrysoeriol O-glucuronic acid), and phytohormone compounds (>173-fold increase of methyl jasmonate) (Fig. 2A; **see****Supporting Information—Table S3**). However, as the stress duration was elapsing, the metabolic processes were impaired leading to the decrease of a large number of metabolites in both cultivars (Fig. 2B; **see****Supporting Information Tables S3****and****S4**). Nonetheless, the tolerant cultivar (0119) could still accumulate the salt stress-responsive metabolites and better maintain the metabolic processes than cultivar 0226, especially after 24 h under stress, a time point which appears to be critical for the salinity stress endurance. After 24 h under stress, as the energy is critically needed, the metabolites from the nucleotide metabolism were strongly accumulated as well as the important stress-related compounds such as vitamin (L-ascorbate), phenolamide (tyramine), coumarin (esculetin), amino acid derivatives (L-tryptophan), phytohormone (abscisic acid (ABA)), polyamine (putrescine), etc. At 72 h under stress, the sensitive cultivar (0226) was overwhelmed and failed to accumulate in optimal levels the majority of the metabolites, which might lead to the death of the seedlings (Fig. 2B).

**Figure 3.**
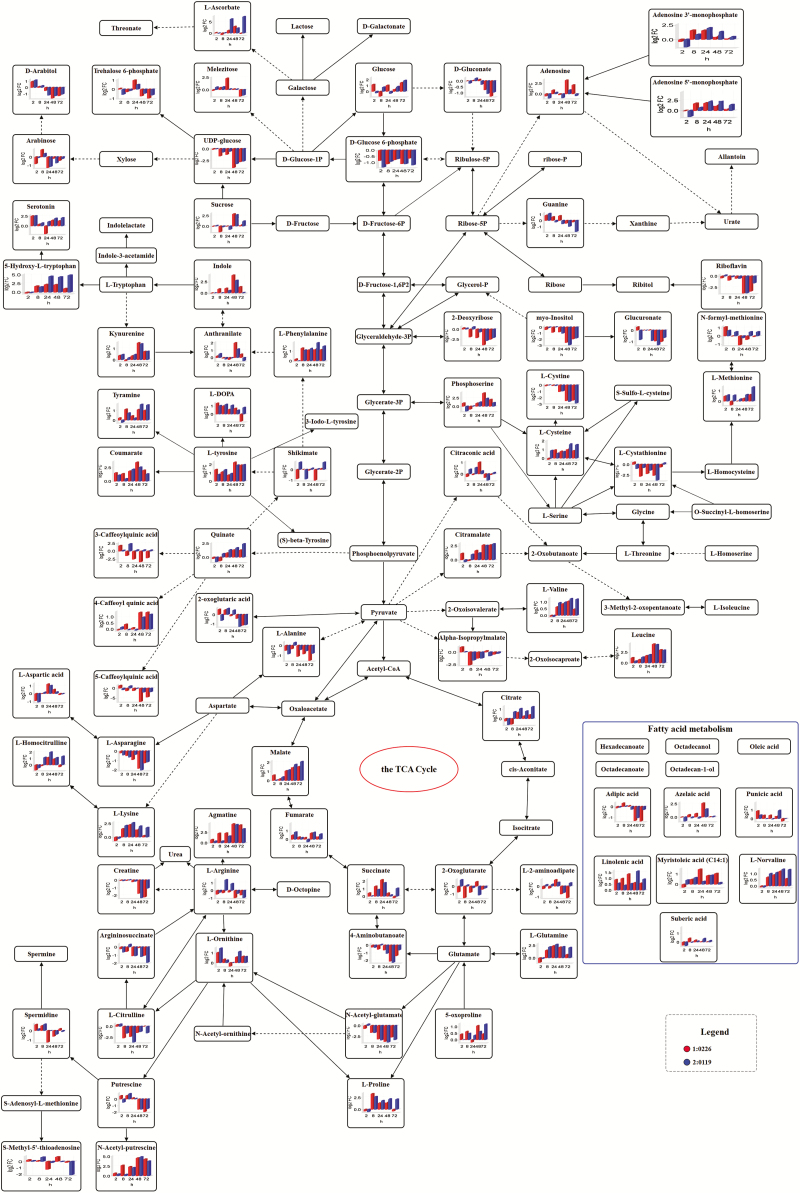
Metabolic pathways of the salt-responsive metabolites in hulless barley. Log_2_ fold change (FC) of significantly changed metabolites mapped to the central metabolic pathways in the leaves of 0119 and 0226 at 2, 8, 24, 48 and 72 h under salt treatment as compared to control treatment. Metabolic pathways were constructed according to the KEGG (http://www.genome.jp/kegg/) metabolic database.

Through the general metabolic pathway mapping, the DAMs in response to salt stress were found to be mainly involved in amino acid metabolism, glycolysis, tricarboxylic acid (TCA) cycle and urea cycle, suggesting that these metabolic pathways play important roles in adaptive response to salt stress in hulless barley (Fig. 3). It also revealed that most of these metabolites, particularly the amino acids, organic acids and osmolytes were highly accumulated mainly during the late stages of salt stress (from 48 to 72 h) in the tolerant cultivar 0119 and a reduced glycolysis activity was maintained until 48 h as compared to the sensitive cultivar 0226. For example, malate, succinate, fumarate, citrate, proline, lysine, linoleic acid, ascorbate, phenylalanine, quinate, etc. were lower in the tolerant cultivar than in the sensitive one at the early stage of salt exposure but, an opposite scenario was observed during the late stage (Fig. 3).

### The core metabolites involved in salt stress response in hulless barley

**Figure 4.**
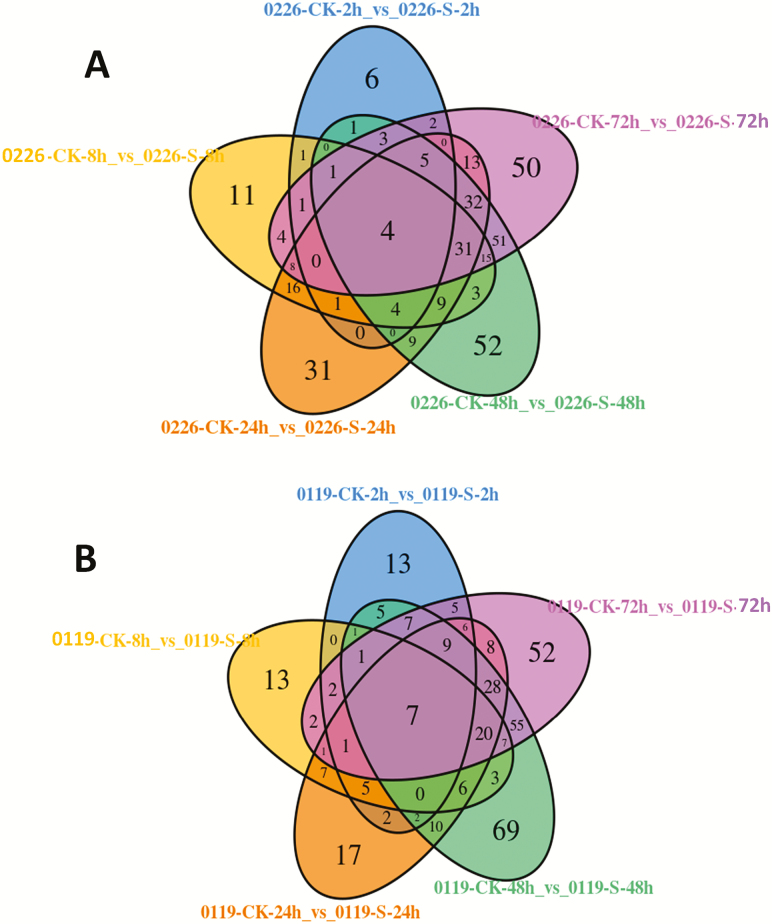
(A) Venn diagram depicting the shared and common DAMs between the time point series under salt stress in 0226; (B) Venn diagram depicting the shared and common DAMs between time point series under salt stress in 0119.

Figure 4A and B present the shared and specific DAMs at the different time points under salinity stresses. It further confirms that specific metabolic adjustments occur at the different time points under salinity stress in hulless barley, though similar metabolites were regulated at the late stages of salinity stress (48–72 h) in both cultivars. We identified 4 and 41 metabolites whose levels significantly changed during all the time points or at four time points, respectively, under salt stress in 0226 **[see****Supporting Information—Table S5****]**. Likewise, the levels of 7 and 31 metabolites were constitutively and significantly changed at five or four time points under stress in 0119, respectively **[see****Supporting Information—Table S6****]**. Among these major salt-responsive metabolites, 13 compounds (piperidine, 5-hydroxy-L-tryptophan, *N*-acetyl-L-glutamic acid, L-saccharopine, L-phenylalanine, 6-methylcoumarin, cinnamic acid, inosine 5′-monophosphate, aminomalonic acid, 6-aminocaproic acid, *N*′,*N*-p-coumaroyl-feruloyl putrescine, *N*-feruloyl tyramine and ABA) were shared by both cultivars representing the core metabolites associated with salt stress responses in hulless barley, regardless of the tolerance levels. In particular, we identified ABA as the only metabolite commonly and constantly changed (increased) during all the time points under salt stress in both cultivars.

### Detecting important salt-tolerance biomarkers in hulless barley

**Figure 5.**
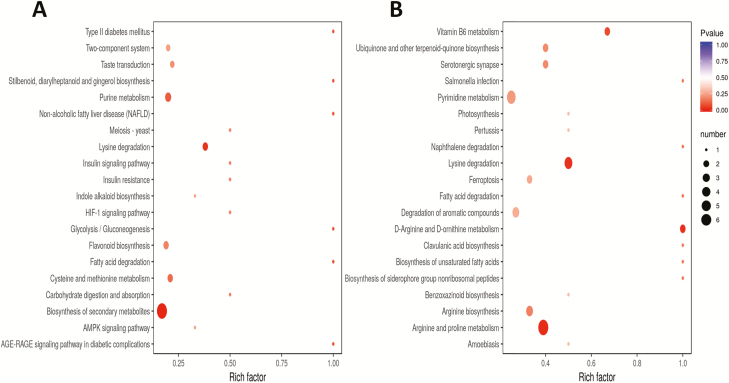
(A) KEGG enrichment analysis of the DAMs between 0119 and 0226 at 2 h under salt stress; (B) KEGG enrichment analysis of the DAMs between 0119 and 0226 at 8 h under salt stress.

**Figure 6.**
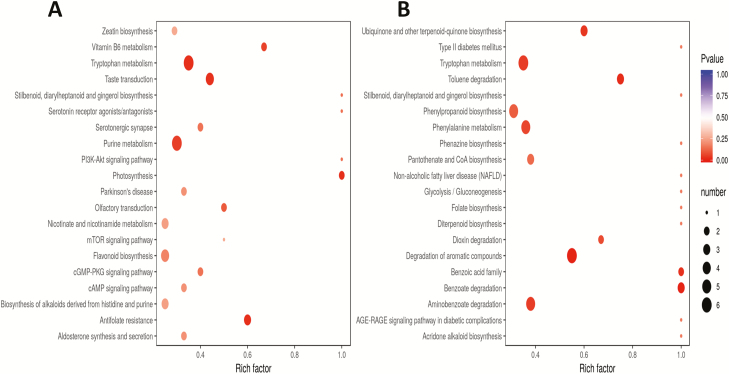
(A) KEGG enrichment analysis of the DAMs between 0119 and 0226 at 24 h under salt stress; (B) KEGG enrichment analysis of the DAMs between 0119 and 0226 at 48 h under salt stress.

**Figure 7.**
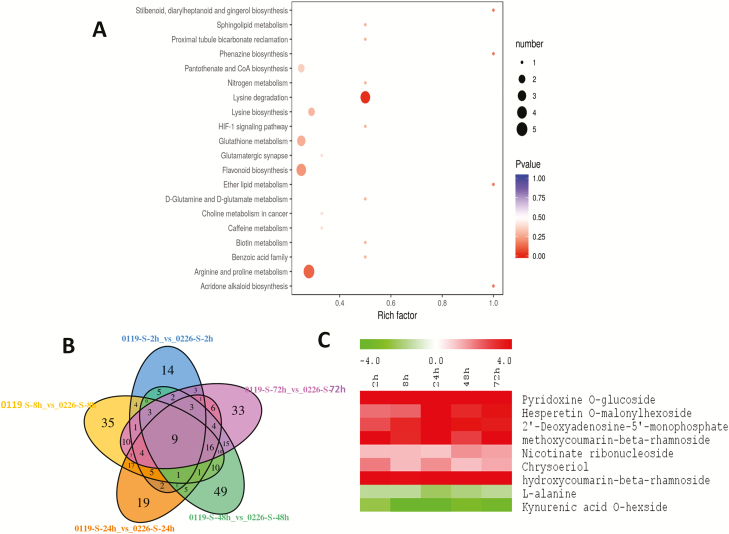
(A) KEGG enrichment analysis of the DAMs between 0119 and 0226 at 72 h under salt stress; (B) Venn diagram depicting the shared and common DAMs between the time point series under salt stress in 0119 compared to 0226; (C) Log_2_ fold change of the constitutively detected DAMs between 0119 and 0226 during salt treatment.

To get an insight into the important metabolites conferring salt tolerance in 0119, we analysed the DAMs between 0119 and 0226 under stress. In total, 295 metabolites were differentially accumulated between the two cultivars at least at one time point under stress **[****see Supporting Information—Table S7****]**. Kyoto Encyclopedia of Genes and Genomes enrichment analysis of the DAMs between the two cultivars at each time point under stress showed a dynamic metabolic adjustment over the stress durations (Figs 5–7). The most enriched biological pathways were purine metabolism, pyrimidine metabolism, arginine and proline metabolism, tryptophan metabolism, flavonoid biosynthesis, phenylpropanoid biosynthesis, glutathione metabolism and lysine degradation. We identified nine metabolites (pyridoxine O-glucoside, L-alanine, hesperetin O-malonylhexoside, kynurenic acid O-hexside, 2′-deoxyadenosine-5′-monophosphate, 4-hydroxy-7-methoxycoumarin-beta-rhamnoside, nicotinate ribonucleoside, chrysoeriol 6-C-hexoside 8-C-hexoside-O-hexoside and 7-hydroxycoumarin-beta-rhamnoside) constitutively and differentially accumulated in 0119 compared to 0226 during all the stress duration (Fig. 7B). These metabolites may represent major biomarkers for salt stress tolerance in hulless barley and interestingly, the majority of these compounds were increased in the tolerant accession, an indication of their positive functions to impart salt tolerance (Fig. 7C).

## Discussion

### Extensive metabolite data for deciphering hulless barley responses and tolerance mechanisms to salinity stress

Metabolites are intermediates or end products of metabolism, playing significant physiological and biochemical roles in plants (Luo 2015). It is estimated that the total number of plant metabolites exceeds 200 000 (Pichersky *et al.* 2006) reflecting their multifaceted functions in plants’ life cycle. In hulless barley, the diversity of metabolites and their functional characterization have not yet been reported. Hulless barley is a resilient crop able to survive several environmental stresses (Li *et al.* 2016); therefore, investigating its metabolic responses to abiotic stress can provide important and novel insights into the tolerance mechanisms to abiotic stress in plants. Among the major abiotic stresses affecting the productivity of major food crops worldwide, salinity is of particular importance but limited progress has been made so far to enhance the crop’s salinity tolerance (Negrão *et al.* 2017). In the present investigation, a rich and diverse set of metabolites was detected for the first time in the leaves of two hulless barley cultivars. Under salt stress, the levels of numerous metabolites were altered, offering an excellent opportunity to identify novel salt-responsive compounds (Shen *et al.* 2016). The temporal metabolic profiling strategy adopted in this study coupled with the comparison of tolerant and sensitive cultivars highlighted that salinity stress-induced metabolic reprogramming is genotype-dependent and time-dependent in hulless barley. Our observations are in perfect agreement with the study of Zhao *et al.* (2014) who observed that metabolites were temporally and genotype-dependently regulated under salt stress in two contrasting rice genotypes. Similarly, Kim *et al.* (2007) revealed that a short-term salt stress affects the phenylpropanoid pathway for lignin and glycine betaine biosynthesis, while a long-term response tends to combine the induction of glycolysis and sucrose metabolism in *A. thaliana*.

### Salinity stress principally disturbs the primary metabolism in hulless barley

Under abiotic stress, the plant metabolism is significantly perturbed (Sanchez *et al.* 2008). The main purpose of studying metabolic changes during stress response is to identify metabolites that allow for the reestablishment of homeostasis and normal metabolic fluxes. We observed that the salt-responsive metabolites in hulless barley were dominated by the primary metabolites. According to Slama *et al.* (2015), primary metabolites are the most important metabolites affected by stress, usually on account of the limited CO_2_ assimilation. In accordance with our findings, salt stress was reported to greatly influence the primary metabolism in various plant species such as maize (Mladenova 1990), rice (Wang *et al.* 2015), barley (Shelden *et al.* 2016), peanut (Cui *et al.* 2018), etc. Particularly, most of the detected salt-responsive primary metabolites belong to the classes of organic acids, nucleotide and its derivates, and amino acid derivatives. It is well-known that organic acid metabolism is of fundamental importance at the cellular level for various biochemical pathways, including energy production, the formation of precursors for amino acid biosynthesis and at the whole plant level in modulating adaptation to abiotic stresses (López-Bucio *et al.* 2000). As photosynthetic intermediates, they are essential to plants (Cockburn 1985) but they also have a potential role as metabolically active solutes for the osmotic adjustment and the balance of cation excess; therefore, their activity is crucial for plant responses to abiotic stresses such as salinity. The nucleotide metabolism is also of great importance to living organisms, as nucleotides are the building blocks of nucleic acids synthesis, energy sources, coenzymes for redox reactions and precursors for the synthesis of primary and secondary products (Moffatt and Ashihara 2002). Similarly, amino acids have various prominent functions in plants, particularly during protein biosynthesis. They represent the building blocks for several other biosynthesis pathways and participate in signalling processes during plant stress response (Hildebrandt *et al.* 2015).

### The sensitive and tolerant cultivars share a core metabolome fundamental to salinity stress responses

Despite their differential tolerance level, sensitive and tolerant genotypes share pools of metabolites which are fundamental to the stress responses. Here, by comparing 0226 and 0119, we uncovered several key metabolites commonly altered in both genotypes under stress. Most of these metabolites were reported to be involved in salt stress response in plants (Sanchez *et al.* 2008). Interestingly, these core metabolites were mainly increased under salt stress, indicating their positive roles in osmotic regulation and salinity endurance in hulless barley. Distinctly, high levels of ABA were constitutively observed in both cultivars throughout the salt stress application. Abscisic acid is described as a key hormone in plant adaptation to abiotic stress such as salt, osmotic and cold (Waśkiewicz *et al.* 2013). One major biochemical change in response to stress is the elevation of ABA levels, which in turn triggers the activation of stress-responsive genes (Wasilewska *et al.* 2008). The ABA signalling pathway can therefore be considered essential to salt stress response in hulless barley.

### The efficient management of glycolysis and energy consumption are the key strategies for tolerating salt stress

In general, the metabolic changes observed in stressed plants varied in their significance according to the tolerant/sensitive phenotypes. Here, we observed that the sensitive cultivar experienced a more pronounced metabolic change as compared to the tolerant one. Similar to our findings, Widodo *et al.* (2009) and Shen *et al.* (2016) observed that within the cultivated and wild barley species, the metabolism under salt stress in the tolerant genotypes was less affected as compared to the sensitive ones. Recently, Fu *et al.* (2018) also demonstrated that rice, which is a salt sensitive species, experiences more serious osmotic stress and metabolic changes than barley, a salt tolerant species. In this study, further investigations into the temporal regulation of the salt-responsive metabolites indicated that the salt tolerance observed in the cultivar 0119 is due to its adaptive reaction at the different stress times. At the early salt stress stages, in contrast to 0226, the tolerant cultivar 0119 tended to save energy by limiting glycolysis activity, nucleotide metabolism and amino acid synthesis, while increasing antioxidant compounds such as flavonoids. And under a long-term exposure to salt stress, the tolerant cultivar 0119 highly supplied energy probably for Na^+^ exclusion from cells and strongly accumulated the metabolites serving as compatible solutes against the osmotic challenge. Recently, Wu *et al.* (2013) also observed that increasing glycolysis and energy consumption during the most stressful time under high salinity stress is a tolerance mechanism in the cultivated barley. In rice, a similar salt stress tolerance mechanism was reported (Zhao *et al.* 2014). Kim *et al.* (2007) also demonstrated that *A. thaliana* resists to a long-term salinity stress by inducing glycolysis and sucrose metabolism, indicating that it is a conserved salt-tolerance mechanism in plants (Sanchez *et al.* 2008).

### Hulless barley provides novel salt-tolerance candidate biomarkers

Candidate metabolites as stress-tolerance biomarkers are useful in breeding programmes and for crop improvement strategies. By analysing the differential salt-affected metabolites in the highly tolerant cultivar 0119 compared to the sensitive one, we anticipated to uncover important salt-tolerance biomarkers. A total of 295 metabolites displayed differential accumulation between the two cultivars under stress, denoting that these compounds are the basis of their contrasting responses to salinity. The metabolites can be grouped into the classes of vitamins, amino acid derivatives, flavonoids, organic acids, nucleotide and its derivates and coumarins, which play essential functions in plant normal metabolism, development and responses to biotic and abiotic stresses (Buer *et al.* 2010; Dell’Aglio *et al.* 2017). Of these diverse metabolites, we pinpointed nine metabolites as the major salt-tolerance biomarkers in hulless barley and their up-accumulation in the tolerant cultivar further highlights their positive functions in salt tolerance. For example, pyridoxine glucoside accumulation was >5-fold in the tolerant cultivar than the sensitive one. Pyridoxine glucoside is a fraction of vitamin B_6_ in plant foods (Gregory *et al.* 1991). Colinas *et al.* (2016) reported that vitamin B_6_ is required for >200 catalytic reactions ranging from hormone biosynthesis to amino acid metabolism in plants. Therefore, the key strategies for plant survival under stress conditions include mechanisms to maintain the homeostasis of B vitamins. Vitamin B_6_ was demonstrated to play a crucial role in protecting cells from oxidative stress because it exhibits antioxidant activity (Vanderschuren *et al.* 2013). Similarly, Huang *et al.* (2013) revealed that abiotic stresses increased the abundance of different B_6_ vitamers, particularly pyridoxine in tobacco plants. Therefore, we deduce that the high concentration of pyridoxine glucoside helps alleviate the osmotic stress caused by salinity stress in hulless barley. Two flavonoid compounds including hesperidin and chrysoeriol were also detected as important biomarkers for salt tolerance in hulless barley. Hesperidin is a plant flavanone (a subclass of flavonoids) naturally and abundantly found in plants such as citrus (Liu *et al.* 2013). The flavanone hesperetin (aglycone) is a glycoside form of hesperidin. Extensive works have shown that flavonoids are accumulated in plants under abiotic stresses and function as absorbing compounds or free radical scavengers to reduce the damages to plants (Pourcel *et al.* 2007; Buer *et al.* 2010). Likewise, chrysoeriol has been shown to possess a potent antioxidant activity (Mishra *et al.* 2003). However, so far, there was no report on the effect of salt stress on hesperitin and chrysoeriol accumulation in plants. Furthermore, their functions in stress tolerance in plants, especially salt stress tolerance are unknown. Besides, many of the salt-tolerance biomarkers detected in our study have not yet been described as associated with abiotic stress response in plants, implying that they may be novel compounds or exclusive salt-tolerance metabolites in hulless barley. Therefore, we propose that future studies may deeply investigate these newly identified compounds, as novel salt-tolerance candidate biomarkers in plants.

## Conclusions

In this report, we generated for the first time extensive metabolic data under progressive salinity stress in two contrasting hulless barley cultivars. Salinity stress affects the metabolism of hulless barley genotypes and the stress-induced metabolic reprogramming is genotype-dependent and time-dependent. Furthermore, salinity stress mainly disturbs the primary metabolism in hulless barley, particularly, compounds from the classes of organic acids, nucleotide and its derivates, and amino acid derivatives. Several metabolites were commonly and differentially altered in both cultivars under salinity stress, representing the core metabolome fundamental to salinity stress responses in hulless barley. Our study highlighted the cardinal importance of ABA in salt stress response signalling in hulless barley. By comparing the metabolic responses triggered by the two contrasting cultivars under salinity stress, it was clear that the efficient management of glycolysis and energy consumption, especially during the most stressful time is the key strategy for tolerating salt stress. Finally, we detected nine salt-tolerance biomarkers, mostly unreported salt-tolerance metabolites, which may help improve salt stress tolerance in plants.

## Availability of Data and Material

The data sets supporting the conclusion of this article are included within the article and the **Supporting Information**. The metabolite quantification data set will be provided upon request addressed to the corresponding author.

## Sources of Funding

This work was supported by the Financial Special Fund (2017CZZX002, XZNKY-2018-C-021).

## Contributions by the Authors

Conceptualization, Z.S., T.N.; methodology, Y.W., X.Z., Q.X.; formal analysis and data curation, X.M., H.Y., D.J.; writing-original draft preparation, Y.W.; writing-review and editing, Z.S., T.N. All authors discussed the results and contributed comments to the manuscript. All authors approved the final version of the manuscript.

## Conflict of Interest

None declared.

## Supporting Information

The following additional information is available in the online version of this article—

**Table S1.** Full list of the metabolites detected in leaves of the two hulless barley cultivars using the widely targeted metabolomics approach.

**Table S2.** List of the differentially accumulated metabolites under salt stress in the tolerant cultivar 0119 and the sensitive cultivar 0226.

**Table S3.** List of the differentially accumulated metabolites at 2, 8, 24, 48 and 72 h under salt stress in the tolerant cultivar 0119.

**Table S4.** List of the differentially accumulated metabolites at 2, 8, 24, 48 and 72 h under salt stress in the sensitive cultivar 0226.

**Table S5.** List of the differentially accumulated metabolites constitutively detected at four and five time points under salt stress in the sensitive cultivar 0226.

**Table S6.** List of the differentially accumulated metabolites constitutively detected at four and five time points under salt stress in the tolerant cultivar 0119.

**Table S7.** List of the differentially accumulated metabolites between the sensitive cultivar 0226 and the tolerant one 0119 at 2, 8, 24, 48 and 72 h under salt stress.

Supplementary Table
